# POU class 2 homeobox associating factor 1 (POU2AF1) participates in abdominal aortic aneurysm enlargement based on integrated bioinformatics analysis

**DOI:** 10.1080/21655979.2021.1990822

**Published:** 2021-10-26

**Authors:** Jinze Meng, Hao Wen, Xintong Li, Boyang Luan, Shiqiang Gong, Jie Wen, Yifei Wang, Lei Wang

**Affiliations:** a Department of Pharmacology, China Medical University, Shenyang, China; b Department of Trauma Center, The First Affiliated Hospital of China Medical University, Shenyang, China; c Department of Vascular Surgery, The First Affiliated Hospital of China Medical University, Shenyang, China; d Key Laboratory of Pathogenesis, Prevention and Therapeutics of Aortic Aneurysm in Liaoning Province, Shenyang, China; e Department of Ultrasonography, Inner Mongolia Baotou City Central Hospital, Baotou, China

**Keywords:** Abdominal aortic aneurysm, signed weighted gene co-expression network analysis, protein-protein interaction, differentially expressed gene analysis, POU class 2 homeobox associating factor 1

## Abstract

Abdominal aortic aneurysm (AAA) is life-threatening, its natural course is progressively sac expansion and rupture. Elegant studies have been conducted to investigate the molecular markers associated with AAA growth and expansion, this topic however, still needs to be further elucidated. This study aimed to identify potential genes for AAA growth and expansion based on comprehensive bioinformatics approaches. Firstly, 29 up-regulated genes were identified through DEGs analysis between large AAA and small AAA in GSE57691. Secondly, signed WGCNA analysis was conducted based on GSE57691 and the green module was found to exhibit the topmost correlation with large AAA as well as AAA, 133 WGCNA hub genes were further identified. Merged gene set including 29 up-regulated DEGs and 858 green module genes was subjected to constructing a PPI network where 195 PPI hub genes were identified. Subsequently, 4 crucial genes including POU2AF1, FCRLA, CD79B, HLA-DOB were recognized by Venn plot. In addition, by using GSE7084 and GSE98278 for verification, POU2AF1 showed potential diagnostic value between AAA and normal groups, and exhibited a significant higher expression level in large AAA samples compared with small AAA samples. Furthermore, immunohistochemistry results indicated up-regulation of POU2AF1 in large AAA samples than small AAA samples, which implies POU2AF1 may be a key regulator in AAA enlargement and growth. In summary, this study indicates that POU2AF1 has great predictive value for the expansion of AAA, and may contribute to the further exploration of pathogenesis and progression of AAA.

## Introduction:

Abdominal aortic aneurysm (AAA) refers to the local dilatation and impairment of the infrarenal abdominal aorta with an aortic diameter ≥30 mm or an enlargement of more than 50%[1]. The natural course of AAA has been demonstrated as aneurysm sac expansion and growth which leads to aneurysm rupture eventually [2]. As reported, ruptured aneurysm is accompanied by a very high mortality rate which is estimated to account for 150,000–200,000 casualties every year globally [3]. Current clinical strategies for managing AAA include open surgery and endovascular aneurysm repair (EVAR) [4,5]. In the past two decades, significant advancements have been made in the treatment of AAA with EVAR [4,5]. Despite lower perioperative comorbidity and mortality rate, the early survival benefit of EVAR was not sustained for the long term because of insufficient durability and higher re-intervention rate [6]. On the other hand, due to inadequate acknowledgment of the pathogenesis of AAA, there is currently no effective medical therapies to block the initiation or limit the growth of aneurysmal sac [3,7]. Based on the studies of various animal models and human samples, AAA is believed to be caused by a combination of genetic and environmental factors that trigger immune-mediated cascades in the aorta [3,8]. Previous studies have demonstrated various genetic markers and molecular pathways involving in the AAA expansion and progression [9]. However, given the complexity of the pathogenesis and irreversible expansion of AAA, the underlying molecular mechanisms and biological processes contributing to aneurysm growth remain to be further elucidated.

With the rapid development of high-throughput sequencing and microarray technologies, a new chapter of understanding the potential molecular mechanisms of AAA has been provided. Previous bioinformatics analyses based on microarray data have revealed differentially expressed genes (DEGs), microRNAs and LncRNAs, which may participate in the molecular basis of AAA initiation and progression [10,11]. However, extracting biologically meaningful information from high-throughout data is still a huge challenge. The emergence of network biology has provided deeper understanding of complex biological systems, and related networks are increasingly used in bioinformatics, such as weighted gene co-expression network analysis (WGCNA) which can detect highly related gene clusters [12]. In most cases, WGCNA was conducted to construct an unsigned co-expression network which ignoring the direction of interconnected nodes. In order to characterize the network biology more precisely, a signed WGCNA network was applied in present study [13].

The aim of this study was to explore the underlying key genes and mechanisms related to the expansion and progression of AAA. Therefore, the present study mainly applied signed WGCNA network, DEG analysis and protein-protein interaction network (PPI) to determine crucial genes related with AAA enlargement. Then, we further performed data validation by microarray datasets and immunohistochemistry experiments. Our findings presented new insights into the key regulator contributing to AAA enlargement and progression, which would provide potential therapeutic targets for clinical application.

## Materials and methods:

### Study design and microarray data acquisition

We downloaded the raw dataset GSE57691 [14] from Gene Expression Omnibus Database [15] (GEO: https://www.ncbi.nlm.nih.gov/geo/) on the National Center for Biotechnology Information (NCBI). The original dataset contains 68 samples, including 20 small AAA samples (aortic diameter: average 53.4 ± 2 mm), 29 large AAA samples (aortic diameter: average 68.4 ± 14.3 mm), 9 AOD samples (aortic diameter: average 19.6 ± 2.6 mm) and 10 normal donor samples (aortic diameter: average 25.7 ± 1.2 mm), which was performed based on GPL10558 Illumina HumanHT-12 V4.0 Expression Beadchip platform. Meanwhile, the raw data of GSE7084 [16] and GSE98278 [17] were downloaded for further data validation.

### Data preprocessing and quality assessment

An automatic pipeline including background correction, log_2_ transformation and quantile normalization was conducted by using lumi [18] package in R software [19]. We re-annotated all microarray probes in GSE57691 with packages AnnotationDbi [20] and illuminaHumanv4.db [21] in R software [19] (version 3.6.1). The average expression value was used when multiple probes corresponded to the same gene. Data quality evaluation was implemented by employing boxplots throughout the preprocessing steps.

### Identification of differentially expressed genes between large AAA and small AAA

The limma [22] package in R software [19] was utilized for DEGs analysis between large AAA group and small AAA group in GSE57691. The false discovery rate (FDR) was calculated for multiple testing correction using the Benjamini and Hochberg method [23]. Up-regulated DEGs in large AAA were screened under the threshold of fold change > 1.5 and FDR < 0.05.

### Signed WGCNA network construction and identification of hub genes

We applied the WGCNA package in R^19^ to construct the signed WGCNA network [24]. The WGCNA algorithm can cluster genes with similar expression patterns into specific modules and correlate modules with interested clinical traits, which is widely used for mining the expression patterns of genes. The process of gene clustering and module allocating can be described as below. Firstly, samples were clustered to exclude outliers based on the average linkage hierarchical clustering method. An appropriate soft threshold power β was then selected by the pickSoftThreshold function to ensure a scale-free network [25]. After that, the similarity matrix based on Pearson’s correlation for all pairs of genes was raised by the soft threshold power β into a signed adjacency matrix. The adjacency matrix could be further transformed into a topological overlap matrix (TOM) followed by the corresponding dissimilarity matrix (1-TOM) calculation. Then, the signed scale free co-expression network was constructed using the average linkage hierarchical clustering method with minModuleSize = 300, deepSplit = 2. Module eigengene (ME) was defined as the principal component of a particular module, which summarizes all genes for a given module into a single characteristic expression profile. To make modules have more capacity, modules were further merged with ME cutheight = 0.2. Pearson’s correlations between MEs and clinical traits were able to be calculated and used to determine interested significant modules. Specifically, module with the highest positive correlation with large AAA was selected as significant module. After that, gene significance (GS: correlation between a given gene and a certain clinical trait) and module membership (MM: correlation between a given gene and a certain module eigengene) were calculated. Genes in the significant module identified from WGCNA analysis were further established and identified as WGCNA hub genes with the criteria of MM > 0.8 and GS > 0.2.

### Construction of protein-protein interaction (PPI) network

Proteins encoded by co-expressed genes might generate tightly interactive biological processes and molecular functions. Here, we constructed PPI network for genes from significant module and up-regulated DEGs based on Search Tool for Retrieval of Interacting Genes/Proteins (STRING: https://string-db.org/) database [26], which is a systematic online tool for accessing interrelationships between proteins. Then, the derived PPI network was visualized and analyzed by MCODE [27] plug-in via Cytoscape [28] (version 3.7.2) software. Genes in the above PPI network with MCODE_Score > 5 were screened as PPI hub genes.

### Functional and pathway enrichment analysis

The Gene Ontology (GO) is a comprehensive resource of computational evolving knowledge in regard to the detailed function of gene sets, which mainly describes biological process, cellular component and molecular function [29]. Kyoto Encyclopedia of Genes and Genomes (KEGG) is a knowledge base for systematic analysis of molecular pathways in terms of the networks of genes [30]. In this study, all genes from up-regulated DEGs, WGCNA hub genes and PPI hub genes were uploaded together to the DAVID [31,32] (https://david.ncifcrf.gov/) database to perform GO and KEGG enrichment analysis. Enriched terms of GO and KEGG pathways were selected with *p* < 0.05.

### Identification of crucial genes for large AAA

The intersection genes among up-regulated DEGs, WGCNA hub genes and PPI hub genes were screened and identified as crucial genes, which might be highly associated with clinical signature.

### Data validation

The GEO datasets GSE7084 and GSE98278 were used in the data validation process. The original dataset GSE7084 contained 8 AAA samples and 7 normal donor samples based on Sentrix Human-6 Expression BeadChip platform. While GSE98278 contained 15 stable small AAA samples and 7 stable large AAA samples based on Illumina HumanHT-12 V4.0 expression beadchip platform. Firstly, the raw data of GSE7084 and GSE98278 were preprocessed and normalized by lumi package. Then, GSE7084 was used to conduct the ROC curves of crucial genes between AAA and normal groups, and the GSE98278 was used to verify the expression levels of crucial genes between large AAA group and small AAA group.

### Acquisition of human tissue samples

The experimental procedures were approved by the Ethics Committee of The First Affiliated Hospital of China Medical University (approval number: 2019–120-2). A total of three human normal infrarenal abdominal aortic wall samples were obtained from organ donors, and five small (diameter<50 mm) and five large (diameter>50 mm) AAA wall samples were obtained from patients who underwent open surgery for AAA in The First Affiliated Hospital of China Medical University from Dec 2019 to Jun 2020. Written informed consents were obtained. Detailed information of included individuals were provided in Supplementary Table 1.

### Immunohistochemistry staining

For immunohistochemistry staining, sections were first deparaffinized and then rehydrated, followed by inactivation of endogenous peroxidase with 3% H_2_O_2_ at room temperature, heat-induced antigen retrieval in an autoclave containing sodium citrate buffer (10 mM, pH 6.0), and blocked with normal goat serum for 30 minutes at room temperature. Afterward, the sections were incubated with primary antibody overnight at 4°C in a humidified chamber, and HRP-conjugated goat anti-rat secondary antibody (1:2000, A0192, Beyotime, China) was incubated for 1 hour at room temperature. Sections were examined with diaminobenzidine (DAB) and stained with hematoxylin before dehydration and microscopic examination. The IHC toolbox plug-in [33] in ImageJ [34] (http://imagej.nih.gov/ij/plugins/ihc-toolbox/) was applied to measure the average intensity of the positive signal for each section. The primary antibody used in the immunohistochemistry experiments was anti-POU2AF1 (1:200, sc-23,932, Santa Cruz, USA).

### Statistical analysis

GraphPad Prism 8.2.1 (GraphPad software, San Diego, CA) was used for statistical analysis and graphing in data validation. Two-tailed Student’s *t* test or Mann Whitney test was used to perform comparison between two groups. *p* < 0.05 was considered to be statistically significant.

## Results:

The present study aims to identify the key genes in regulating AAA enlargement and progression. For this purpose, integrated bioinformatics approaches including DEGs analysis, signed WGCNA and PPI network analysis were implemented based on GSE57691. Results showed POU2AF1, FCRLA, CD79B and HLA-DOB were statistical up-regulated in large AAA samples compared to small AAA samples. Moreover, data validation based on GSE57691 and GSE7084 verified these four genes were all up-regulated in AAA samples compared to normal samples, however, further bioinformatics analysis based on GSE57691 and GSE98278 showed only POU2AF1 was consistent statistically significance in large AAA samples compared to small AAA samples. Subsequent immunohistochemistry results also confirmed that POU2AF1 were highly associated with AAA and aneurysm enlargement. Detailed results were shown below.

### Data preprocessing

**Figure 1.**
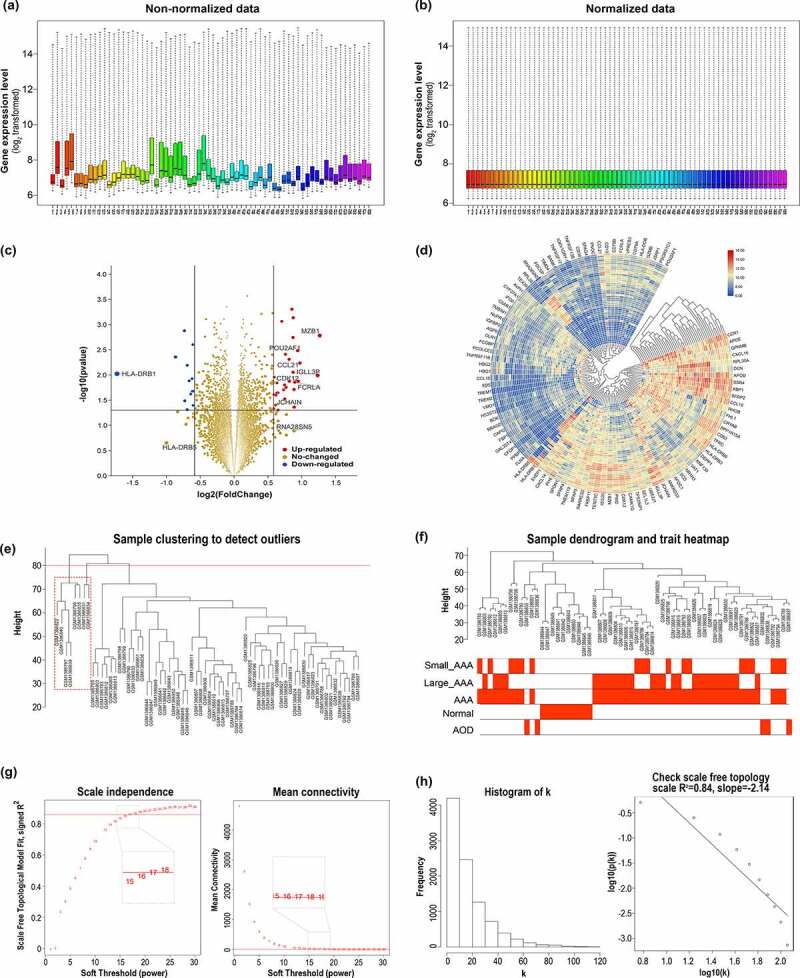
Data preprocessing, DEGs identification in GSE57691, and sample selection and determination of soft-thresholding power β in the signed WGCNA analysis. (a) Non-normalized data: the boxplot shows an obvious deviation across samples before data preprocessing. (b) Normalized data: the boxplot indicates that the normalized data are eligible for subsequent analysis. The gene expression level are represented as log2 transformed. (c) The volcano plot of all genes, the red dots represent upregulated genes, and the blue dots represent all the downregulated genes. (d) Heatmap of top 100 expression level changed genes. (e) Sample clustering to detect outliers. (f) Sample dendrogram and trait heatmap. (g) Analysis of the scale-free fit index (left) and the mean connectivity (right) for various soft-thresholding powers. (h) Histogram of connectivity k distribution (left) and checking the scal-free topology when soft-thresholding power β = 17

The raw data of GSE57691 were downloaded and an obvious deviation was identified between samples in the original dataset, as shown in Figure 1a. Background correction, log_2_ transformation and quantile normalization were performed and the boxplot was reestablished in Figure 1b, which indicated that the normalized data were eligible for further analysis.

### Identification of differentially expressed genes between large AAA and small AAA

DEGs analysis between large AAA and small AAA yielded a total of 42 DEGs, including 29 up-regulated genes and 13 down regulated genes. The volcano plot and a brief heatmap for DEGs analysis were shown in Figure 1c and d, respectively.

### Signed WGCNA network construction and identification of hub genes

Eight samples were recognized as outliers and were excluded from the subsequent analysis (Figure 1e). Thus, remaining 60 samples (18 small AAA samples, 27 large AAA samples, 5 AOD samples and 10 normal donor samples) with clinical information were included for WGCNA analysis (Figure 1f). A total of 9521 genes in the top 50% of variances were included in WGCNA. In this study, the power of β = 17 (R [2]=0.84) was selected as the soft threshold to guarantee a scale-free network distribution (Figure 1g,h).

**Figure 2.**
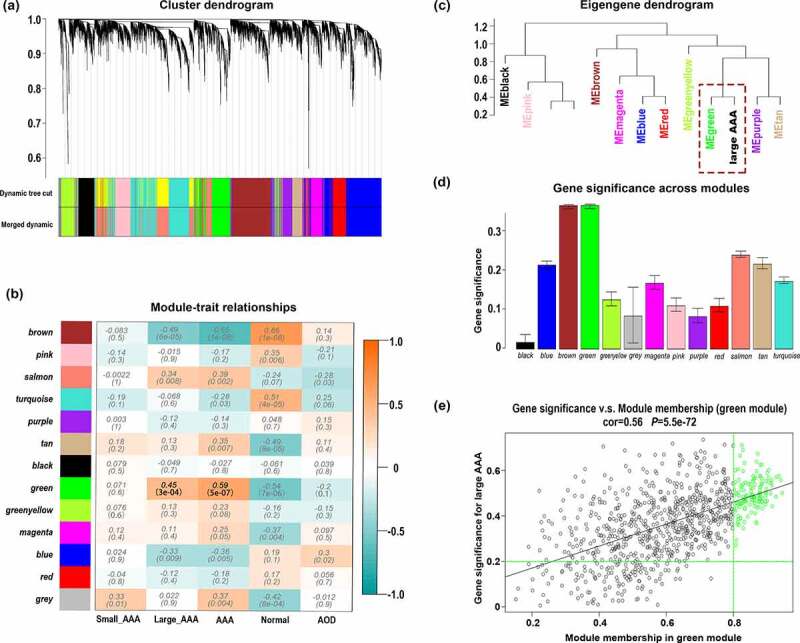
Signed WGCNA analysis for determination of modules and hub genes associated with clinical traits of large AAA. (a) Dendrogram of all genes in GSE57691 clustered based on the dissimilarity measure (1-TOM). (b) Heatmap of the correlation between MEs and clinical traits. (c) Eigengene dendrogram. (d) Distribution of average GS and errors across the modules detected by signed WGCNA analysis. (e) Scatter plot of GS and MM for genes in the green module, the green circles represent WGCNA hub genes with GS > 0.2 and MM > 0.8. TOM: topological overlap matrix; ME: module eigengene; GS: gene significance; MM: module membership; AOD: aortic occlusive disease; AAA: abdominal aortic aneurysm; WGCNA: weighted gene co-expression analysis

A total of 13 distinct modules were identified (Figure 2a). To better reveal the biological significance of the 13 identified modules, we correlated the 13 MEs with the traits of interest and tried to figure out the most significant correlation. According to the module traits heatmap (Figure 2b), the green module (858 genes) appeared to have strong positive correlation with large AAA (r = 0.451, *p*= 3*10^−^[4]) as well as AAA (r = 0.59, *p* = 5*10^−^[7]). In the eigengene dendrogram (Figure 2c), the green module was clustered with large AAA, which also indicates a strong positive relationship between green module and large AAA. Consistent with the above conclusions, the green module had the highest GS in large AAA, which also indicates a strong relationship between green module and large AAA (Figure 2d).

Furthermore, we identified 133 hub genes with GS > 0.2 and MM > 0.8 in green module based on the scatter plots of GS and MM for large AAA (Figure 2e). Therefore, we would mainly focus on 133 hub genes in the following processes since these genes may indicate clinical signature more accurately.

### PPI network construction

**Figure 3.**
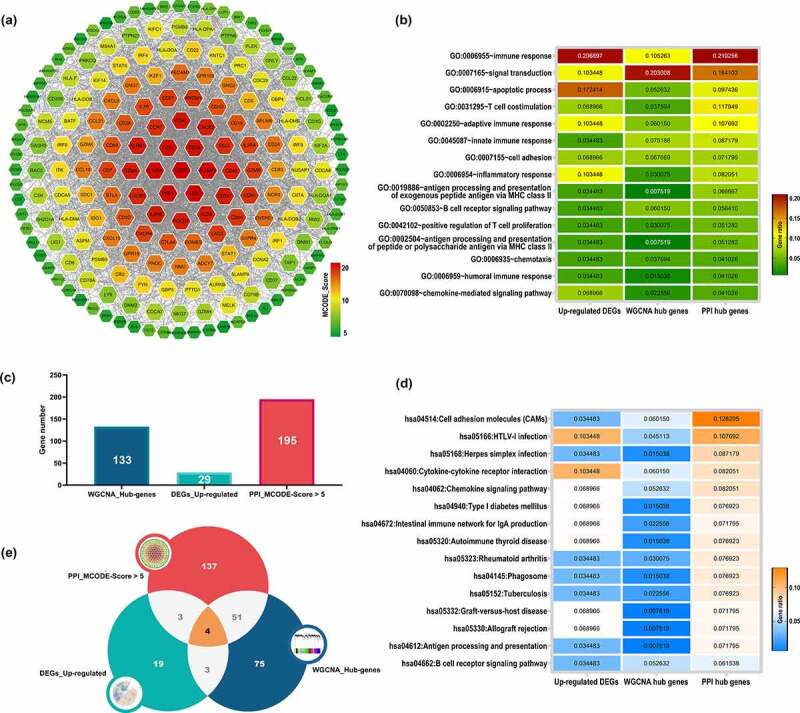
PPI network construction and functional annotation. (a) PPI network construction based on the union gene set of 29 up-regulated genes and 858 green module genes, nodes color represent the level of MCODE_Score. Functional annotation for WGCNA hub genes, DEG upregulated genes and PPI hub genes: (b) Top 15 enriched GO_BP terms for three groups of genes. (d) Top 15 enriched KEGG pathways for three groups of genes; Gene ratio in every group were represented by color. (e) Histogram of gene numbers of WGCNA hub genes, DEG upregulated genes and PPI hub genes. (f) Venn plot of intersection gene set among three groups of genes. WGCNA: weighted gene co-expression analysis; DEGs: differentially expressed genes; PPI: protein- protein interaction; PPI: protein- protein interaction; GO: Gene Ontology; BP: biological process; KEGG: Kyoto Encyclopedia of Genes and Genomes

Here, we constructed PPI network based on the union gene set (865 distinct genes) of green module and up-regulated DEGs via STRING database. Eventually, a PPI network with 637 nodes and 4323 edges was obtained. After analyzed with MCODE plug-in, a total of 195 genes were selected with the cutoff criteria of MCODE_Score > 5, which were recognized as PPI hub genes. The interaction network for 195 PPI hub genes were illustrated in Figure 3a.

### Functional annotation and enrichment analysis

We conducted GO and KEGG functional enrichment analysis in DAVID database. The union gene set of up-regulated DEGs (29 genes), WGCNA hub genes (133 genes) and PPI hub genes (195 genes) was subjected as input. As a result for biological processes (Figure 3b), three groups of genes were significantly enriched in immune system and inflammatory activities, including immune response, signal transduction, apoptotic process, T cell costimulation, adaptive immune response, innate immune response, cell adhesion, inflammatory response, antigen processing and presentation of exogenous peptide antigen via MHC class II, B cell receptor signaling pathway, positive regulation of T cell proliferation, antigen processing and presentation of peptide or polysaccharide antigen via MHC class II, chemotaxis, humoral immune response and chemokine-mediated signaling pathway. As for the KEGG pathway enrichment analysis (Figure 3d), three groups of genes were mainly associated with cell adhesion molecules, cytokine-cytokine receptor interaction, chemokine signaling pathway, intestinal immune network for IgA production, autoimmune thyroid disease, phagosome, graft-versus-host disease, allograft rejection, antigen processing and presentation and B cell receptor signaling pathway.

### Identification of crucial genes for large AAA

In this study, we identified 29 up-regulated DEGs and 133 WGCNA hub genes highly associated with large AAA status. Subsequently, 195 PPI hub genes were screened through PPI network construction (Figure 3c). In order to manifest the genetic characteristics of large AAA more accurately, we defined the intersection genes of these three gene sets as crucial genes, which generated 4 genes include POU2AF1, FCRLA, CD79B and HLA-DOB, as illustrated by the Venn diagram (Figure 3e). These 4 crucial genes should be highly associated with large AAA status which may need further validation.

### Validation of crucial genes

**Figure 4.**
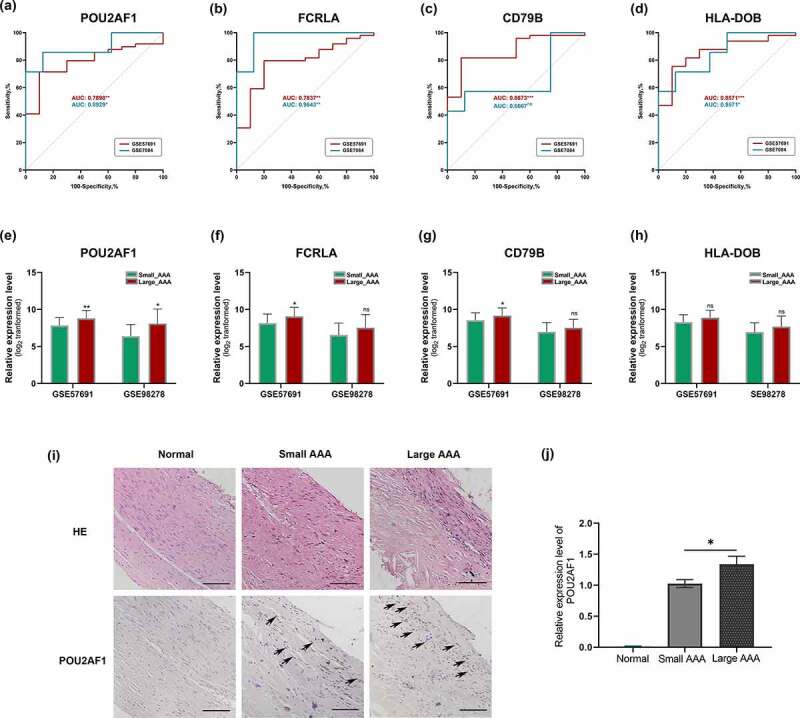
Verification of POU2AF1. (a-d) ROC curves for POU2AF1, FCRLA, CD79B and HLA-DOB of AAA based on GSE57691 and GSE7084, respectively. (e-h) Expression levels of POU2AF1, FCRLA, CD79B and HLA-DOB between large AAA and small AAA based on GSE57691 and GSE98278, respectively. (i) Representative images of H&E and immunohistochemistry staining for POU2AF1 in the aortic walls of normal aorta (left), small AAA (middle) and large AAA (right). (j) Measurements of POU2AF1 per cross-section were shown for the three groups of samples. *p < 0.05; Mann Whitney test was used to evaluate the statistical significance of differences

We used GSE7084 and GSE57691 to conduct the ROC analysis for crucial genes in terms of AAA status. The area under curve (AUC) and corresponding *p*-value for each gene was calculated. As Figure 4a illustrated, POU2AF1 showed significant diagnostic value both in GSE7084 (AUC: 0.8929, p < 0.01) and GSE57691 (AUC: 0.7898, p < 0.05). Similar results were achieved for FCRLA (AUC: 0.9643, p < 0.01 and 0.7837, p < 0.01, respectively) (Figure 4b) and HLA-DOB (AUC: 0.8671, p < 0.05 and 0.8571, p < 0.001, respectively) (Figure 4d). However, CD79B did not show significant diagnostic value in GSE7084 (AUC: 0.6607, p > 0.05) (Figure 4c).

To further verify the expression levels of crucial genes between large AAA and small AAA, we used GSE98278 and GSE57691 for further analysis. In summary, POU2AF1 was significantly up-regulated in large AAA samples both in GSE98278 (8.077 ± 1.976 vs 6.386 ± 1.580, p < 0.05) and GSE57691 (8.787 ± 1.085 vs 7.848 ± 1.047, p < 0.01) (Figure 4e). However, FCRLA, CD79B and HLA-DOB were failed to show statistically significant differences between large AAA and small AAA in GSE98278 (Figure 4f-h). Above results indicated that POU2AF1 was significant up-regulated in large AAA and had a potential diagnostic value in AAA, which might act as a novel biomarker in the AAA enlargement process.

Next, we verified the expression of POU2AF1 in AAA of different diameters using human samples by immunohistochemistry experiments (Figure 4i, j). The results showed that POU2AF1 was significantly up-regulated in large AAA samples than small AAA samples, while barely expressed in normal aortic tissues.

## Discussion:

In this current study, DEGs analysis between large AAA and small AAA groups generated 42 DEGs, including 29 up-regulated genes and 13 down-regulated genes (Figure 1). WGCNA algorithm was used to achieve a signed weighed gene co-expression network and identify significant gene module featuring the highest correlation with large AAA. In the results of WGCNA analysis, 13 modules were detected and the green module (858 genes) exhibits the topmost relationship with large AAA as well as AAA (Figure 2). In the green module, a total of 133 WGCNA hub genes were further identified based on the GS and MM parameters (Figure 2). Merged gene set including 29 up-regulated DEGs and 858 genes in green module was subjected to constructing a PPI network in which 195 PPI hub genes were screened with MCODE_Score >5 (Figure 3). In order to manifest the gene characteristics of large AAA more precisely, 4 crucial genes POU2AF1, FCRLA, CD79B, HLA-DOB were recognized by converging 29 up-regulated DEGs, 133 WGCNA hub genes and 195 PPI hub genes. Among them, POU2AF1 showed potential diagnostic value between AAA and normal groups and exhibited a significant higher expression level in large AAA samples compared with small AAA samples (Figure 4), which indicates POU2AF1 may serve as a key regulator in AAA enlargement and growth.

POU class 2 homeobox associating factor 1 (POU2AF1, also known as OCA-B, OBF-1 and BOB-1) is previously recognized as a B lymphocytes specific coactivator of octamer-binding transcription factors, OCT1 and OCT2, to regulate immunoglobulins expression and additional immune related genes [35,36]. It is reported that POU2AF1 participates in the immune and inflammatory reactions associated with atherosclerosis [36,37].However, current evidence about the biological function of POU2AF1 in AAA is limited. Study by IJpma [38] revealed POU2AF1 act as a transcription factor in AAA but the underlying mechanisms are unknown.

POU2AF1 has no intrinsic DNA-binding activity but can specifically recognize and bind to the POU domain of OCT1 and OCT2, which plays a vital role in B lymphocytes activation and maturation and is required for the formation of germinal centers [39–42]. Involvement of B lymphocytes and associated cytokines and immunoglobulins in AAA pathogenesis and development have been implicated in previous studies [43]. Koch et al. [44]. found a significant higher level of B lymphocytes infiltration in the adventitia of AAA compared with occlusive aortas and normal aortas. Further studies confirmed that not only B lymphocytes but also lymphoid follicles containing B cell aggregated germinal centers in the adventitia of AAA wall [45,46]. Ocana et al. [47]. identified AAA infiltrating B lymphocytes as activated memory cells with homing properties which also give rise to the formation of lymphoid structures and germinal centers within AAA wall. Numerous studies have also shed light on the correlation of B lymphocytes produced immunoglobulins such as IgG and IgE with AAA [48,49].Moreover, elevated immunoglobulins activate the complement cascade through the classical pathway, the lectin pathway and the alternative pathway, which was reported to contribute to the membrane attack complex and aggravate the aortic inflammatory responses [50–53]. Furthermore, evidence showed that POU2AF1 accompanied OCT2 transcriptionally regulate IL-6 expression in B cells during antiviral responses [54], but the direct transcriptional regulation of POU2AF1 for other B lymphocytes specific cytokines are less elucidated and need to be further investigated. In this study, we identified POU2AF1 as a crucial gene in large AAA compared with small AAA by integrated bioinformatics analysis, also, we found that B cell signaling pathway was significantly enriched in the GO and KEGG analysis, which in together suggest that POU2AF1 might function as a transcriptional coactivator in modulating B lymphocytes related biological processes in AAA expansion and growth.

Although POU2AF1 is well acknowledged to be expressed and functioned in B lymphocytes development, evidence showed that its expression is also inducible in T lymphocytes [55]. T lymphocytes are heterogeneous which have initially been classified as CD4+ and CD8 + T lymphocytes. The CD4 + T lymphocytes have been found to be the predominant cell type in AAA [56], which is consist with the T cell associated signaling pathways and MHC class II mediated antigen processes in our GO and KEGG analysis, since CD4 + T lymphocytes recognize antigens presented by MHC II class molecules while CD8 + T lymphocytes recognize antigens presented by MHC I class molecules. CD4 + T lymphocytes can be further subdivided into Th1, Th2, and Th17 and Treg lymphocytes. However, the definite roles of different CD4 + T lymphocytes subtypes in AAA pathogenesis and expansion are conflicting due to the differences in technical measurement, animal models, and the disease state at which aneurysm samples are obtained [57]. Hence, this study is not going to discuss the underlying functions of different CD4 + T lymphocytes subtypes in AAA initiation and progression. In general, it is undisputed that different subtypes of CD4 + T lymphocytes participate in the extracellular matrix remodeling and aortic inflammation through their diverse profiles of secreted cytokines. Brunner et al. [57,58] reported that POU2AF1 directly controlled the IFN-γ and IL-2 (Th1 cytokines) promoter activities while indirectly interfered Th2 cytokines production, which suggests POU2AF1 balanced the Th1 versus Th2 mediated immunity responses. Yosef et al. [59]found that POU2AF1 promotes IL-17 secretion during Th17 differentiation, which was further proved in another study revealing that POU2AF1 enhances IL-17 expression through interaction with RORγt [60]. Furthermore, other studies showed that POU2AF1 is critical for CD4+ memory T cell formation [61] and can regulate spreading manner of follicular helper T cells over the body from local sites during immune responses [62]. Taken together, POU2AF1 might contribute to AAA initiation and expansion by modulating different subtypes of CD4 + T lymphocytes.

Except for the distinct biological function of POU2AF1 in regulating B lymphocytes and T lymphocytes, respectively, studies have also addressed POU2AF1 as a facilitator of B and T lymphocytes collaboration during humoral immune responses [63]. Karnowski et al. [54]found that IL-6 produced by follicular B lymphocytes was necessary and important to induce IL-21 from CD4 + T lymphocytes. Moreover, POU2AF1 might not be restrictively expressed in lymphocytes, evidence can be found from a recent study that POU2AF1 also functions in the human airway epithelium to regulate expression of host defense genes [64]. Given that, we can not preclude the possibility of POU2AF1 expression in other AAA related cell types since the microarray data in this study was achieved from full-thickness tissue sections of aortas.

In addition to certain individual genes, immune and inflammatory associated cells and signaling pathways also contribute to AAA enlargement and progression. In past decades, studies have implicated crucial roles of various inflammatory cells, including T cells, B cells, macrophages, dendritic cells, neutrophils, and mast cells, etc, as well as their intercellular communications and cytokines secretion abilities in AAA initiation and progression [65]. Moreover, complex cellular signaling pathways, such as NF-κB [66], TGF-β[67], MAPK [68], Notch [69] and IL-6 [70] signaling contribute to AAA progression. Results of pathway enrichment analysis in our study also highlighted the significance of inflammatory cells and their intercellular communication signaling pathways, such as T cell costimulation, B cell receptor signaling pathway, cell adhesion molecules, cytokine-cytokine receptor interaction and chemokine signaling pathway. As mentioned above, POUAF1 might act as a transcription factor and participate in B cell and T cell stimulation as well as their related signaling pathways, however, the exact underlying mechanisms need to be further elucidated.

Several studies have also investigated the key genes and related signaling pathways in AAA by bioinformatics analysis. Siwei et al. [71]. revealed conservative co-expression modules and miRNA-genes network in intracranial, abdominal, and thoracic aneurysms. Results showed CCR7, TNF and CXCR4 related miRNA-genes network were preserved in all three kinds of aneurysms, which highlighted the common molecular networks shared by aneurysmal diseases. Moreover, Kan et al. [71]. performed WGCNA analysis and found crucial hub-genes associated with AAA progression. They further predicted potential drug candidates to prevent AAA expansion which would definitely provide guidance for future pharmacotherapy of AAA. In another research, Xie et al. [72]. showed the expression level of RPL21 or RPL7A combined with IL6 has significant diagnostic value for AAA, which provided new insights into the underlying mechanisms of AAA progression. Zhang et al. [73]. revealed several proteases involved in the formation and progression of AAA by next-generation sequencing of the whole transcriptome of Angiotensin II-treated ApoE−/− mice. Li et al. [74]. comprehensively analyzed profiles of infiltrated immune cells in AAA tissues and their associated marker genes, which provided insights into the underlying mechanisms of AAA formation and progression regarding to immune infiltration. The current study focused on exploring the up-regulated key genes in AAA enlargement and progression, thus, microarray datasets with detailed sample diameter information were included. Instead of using merely single algorithm, we implemented integrated bioinformatics approaches including DEG analysis, signed WGCNA analysis and PPI network construction. Compared with another study conducting WGCNA analysis in AAA [75], we mainly focused on exploring the up-regulated key genes and targets in AAA enlargement, to achieve this purpose, we constructed a signed WGCNA network rather than commonly conducted unsigned WGCNA network.

Although the results of microarray based integrated bioinformatics analysis are instructive, our study has several limits. Further studies focusing on elaborating the precise cell specificity and molecular mechanisms of POU2AF1 in AAA initiation and progression are needed. Apart from that, bioinformatic databases used in this study such as STRING [26] and DAVID [31] may be not comprehensive and timely-updated, which may cause information bias in our study.

## Conclusion:

In conclusion, we identified the pivotal role of POU2AF1 in the pathogenesis and expansion of AAA by integrated bioinformatics approaches and experimental validation. Further investigations are recommended to validate and elucidate the detailed biological function and molecular mechanisms of POU2AF1 in AAA initiation and progression.

## Supplementary Material

Supplemental Material

## Availability of data and materials:

The datasets used and/or analyzed in this study are available from the Gene Expression Omnibus (GEO) database.
