# Supplementary material for: POU class 2 homeobox associating factor 1 (POU2AF1) participates in abdominal aortic aneurysm enlargement based on integrated bioinformatics analysis
Source: Bioengineered. 2021 Oct 26;12(1):8980–93. doi: 10.1080/21655979.2021.1990822 (PMC8806937; doi:10.1080/21655979.2021.1990822)
Supplement: Supplemental Material [file KBIE_A_1990822_SM0630.doc]

| **Group** | **No.** | **Gender** | **Age** | **Maxium aortic diameter** | **Smoke** | **Hypertension** | **Diabetes** | **Coronary Heart Disease** | **Stroke** | **Respiratory Disease** | **Renal disease** | **Cause of death** |
| --- | --- | --- | --- | --- | --- | --- | --- | --- | --- | --- | --- | --- |
| Normal Donor | 1 | male | 62 | 28mm | Y | Y | N | N | N | N | N | Traffic accident |
|  | 2 | female | 61 | 24mm | N | N | N | N | N | N | N | Traffic accident |
|  | 3 | male | 55 | 25mm | Y | N | N | N | N | N | N | Traffic accident |
| Small  AAA | 4 | male | 68 | 45mm | N | Y | N | N | N | N | N | / |
|  | 5 | female | 81 | 42mm | N | Y | Y | N | N | N | N | / |
|  | 6 | male | 67 | 47mm | Y | Y | N | N | N | N | N | / |
|  | 7 | male | 74 | 45mm | Y | Y | N | Y | N | N | Y | / |
|  | 8 | male | 78 | 47mm | Y | Y | N | N | N | Y | N | / |
| Large AAA | 9 | male | 77 | 62mm | Y | N | N | N | N | N | N | / |
|  | 10 | male | 78 | 56mm | Y | Y | N | N | N | N | N | / |
|  | 11 | male | 67 | 68mm | Y | Y | Y | Y | N | N | Y | / |
|  | 12 | male | 64 | 75mm | Y | Y | N | N | Y | N | N | / |
|  | 13 | female | 76 | 83mm | N | Y | N | N | N | Y | N | / |

**Supplementary table 1.** Detailed information of included individuals in this study.
